# Photonic Dirac waveguide in inhomogeneous spoof surface plasmonic metasurfaces

**DOI:** 10.1515/nanoph-2024-0200

**Published:** 2024-07-11

**Authors:** Yuting Yang, Juyi Zhang, Bin Yang, Shiyu Liu, Wenjie Zhang, Xiaopeng Shen, Liwei Shi, Zhi Hong Hang

**Affiliations:** School of Materials and Physics, 12392 China University of Mining and Technology, Xuzhou 221116, China; State Key Laboratory of Millimeter Waves, Southeast University, Nanjing 210096, China; School of Physical Science and Technology & Collaborative Innovation Center of Suzhou Nano Science and Technology, Soochow University, Suzhou 215006, China; Institute for Advanced Study, Soochow University, Suzhou 215006, China

**Keywords:** metasurface, spoof surface plasmon, topological photonics, Dirac waveguide, chiral Landau level

## Abstract

The metamaterial with artificial synthetic gauge field has been proved as an excellent platform to manipulate the transport of the electromagnetic wave. Here we propose an inhomogeneous spoof surface plasmonic metasurface to construct an in-plane pseudo-magnetic field, which is generated by engineering the gradient variation of the opened Dirac cone corresponding to spatially varying mass term. The chiral zeroth-order Landau level is induced by the strong pseudo-magnetic field. Based on the bulk state propagation of the chiral Landau level, the photonic Dirac waveguide is designed and demonstrated in the experimental measurement, in which the unidirectionally guided electromagnetic mode supports the high-capacity of energy transport. Without breaking the time-reversal symmetry, our proposal structure paves a new way for realizing the artificial in-plane magnetic field and photonic Dirac waveguide in metamaterial, and have potential for designing integrated photonic devices in practical applications.

## 1 Introduction

The artificial synthetic gauge field has attracted intense research interests in many systems. By applying the strain to the graphene, the deformation of the lattice and the shift of Dirac points give rise to the effective gauge field of vector potential, and the synthetic pseudo-magnetic field perpendicular to the graphene is introduced [1], [2], [3], [4], [5], [6]. The pseudo-magnetic field functioning as an external genuine magnetic field leads to the quantization of Landau levels and quantum-Hall-like effects. Similar to the strained graphene, the synthetic gauge field also can be generated in the sonic crystals and photonic crystals by artificially deforming the lattice shape and structure parameters [7], [8], [9], [10], [11], [12], [13], [14], [15], [16], [17], [18], [19], [20], [21].

In the two-dimensional Dirac material, the discretized platforms of Landau levels are all flat, and the transport of edge states between adjacent Landau levels distributes on the boundary. However, the chiral zeroth-order Landau level is dispersive linearly in the three-dimensional Weyl semimetal, and supports unidirectional transport based on the chirality of Weyl points and the direction of magnetic field [22], [23], [24]. Compared with the edge state localized on the edge or interface of the topological insulator [25], [26], [27], [28], the chiral Landau level is bulk state and propagates inside the system. The chiral anomaly bulk state can be induced by imposing boundary condition in the two-dimensional Dirac system [29], and its direction of transport can be modulated by periodically staggered potential [30]. Different from the perpendicular pseudo-magnetic field generated by the shift of Dirac points, the in-plane pseudo-magnetic field can be obtained by spatially varying mass term. This engineered synthetic gauge field has been utilized to trap the photonic mode in a photonic Dirac cavity [31], guide the topological edge state in a Dirac waveguide of a nanophotonic metasurface [32], [33], and realize the chiral Landau level in a two-dimensional photonic crystal [34].

In this work, we experimentally investigate the property of the synthetic gauge field in the spoof surface plasmonic (SSP) metasurface, which supports the localized surface wave that propagates on the interface between the metallic and dielectric materials [35], [36], [37], [38], [39], [40]. The pseudo-magnetic field is induced by spatially varying the inhomogeneous mass term in the SSP metasurface which belongs to the quantum valley-Hall topological system [41], [42], and gives rise to the chiral zeroth-order Landau level. The bulk state of the chiral Landau level is used to realize the photonic Dirac waveguide. We experimentally demonstrate the propagating property of the straight and Ω-shaped Dirac waveguides, and obtain the beam splitter composed of the bulk mode of the chiral Landau level and the edge state of the topological valley-Hall structure.

## 2 Results and discussion

**Figure 1:**
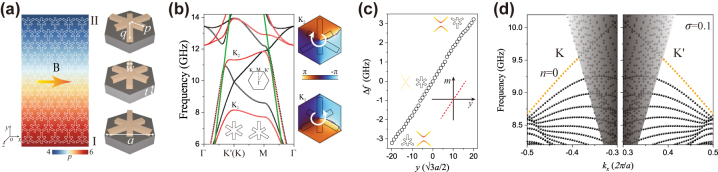
In-plane pseudo-magnetic field in an inhomogeneous SSP metasurface. (a) Schematic of an inhomogeneous SSP metasurface composed metallic snowflake pattern deposited on a dielectric substrate. The structure parameters are the lattice constant *a* = 12 mm, the thickness of dielectric substrate *t* = 1 mm, and the width of metallic pattern *w* = 1.5 mm. The length of three arms of the metallic snowflake pattern are linearly modulated from *p* = 6 mm (bottom layer) to *p* = 4 mm (top layer) represented by the geometry and color bar. (b) Band diagram of the unit cell with a degenerated Dirac point (black lines) and a band gap (red lines), corresponding to the SSP metasurface with *p* = *q* = 5 mm (*C*_6_ symmetry) and *p* = 6 mm, *q* = 4 mm (*C*_3_ symmetry), respectively. Inset: the first Brillouin zone. The phase distributions reveal *K*_1_ and *K*_2_ states have opposite chirality. (c) Bandwidth of *K* points as a function of *y* coordinate for a gradient SSP metasurface. The SSP structure has 41 layers (unit cells) along *y* direction, corresponding to the gradient *σ* = 0.05 mm. Inset: the effective mass term has a linear relation with *y* coordinate. (d) Quantized Landau levels induced by the pseudo-magnetic field corresponding to the gradient *σ* = 0.1 mm and *L* = 21 layers.

The designed inhomogeneous SSP metasurface is illustrated in Figure 1(a), composed of the metallic snowflake pattern deposited on a dielectric substrate. The substrate has a relative permittivity *ɛ* = 2.65 and the metallic pattern can be considered as the boundary condition of the perfect electric conductor in the microwave range. The SSP metasurface consists of the triangular lattice, in which the lattice constant is *a* = 12 mm and the thickness of the substrate is *t* = 1 mm. When the snowflake pattern possesses *C*_6_ symmetry with *w* = 1.5 mm and *p* = *q* = 5 mm, the degenerated Dirac cones appear at *K* and *K*′ points in the calculated TM band (*E*_*z*_ along the *z* axis), as shown in Figure 1(b). The light line is indicated by the green lines, in which the electromagnetic wave radiates into the air. According to the ***k*** **⋅** ***p*** theory [39], [43], [44], the effective Hamiltonian of the SSP metasurface around *K*/*K*′ valleys is

$$H_{K/K^{\prime }}=\nu_{D}\left(\Delta k_{x}\sigma_{x}\pm \Delta k_{y}\sigma_{y}\right)+m\left(\vec{r}\right)\nu_{D}^{2}\sigma_{z}$$

where *v*_*D*_ is the group velocity at the Dirac point, *σ*_*x*,*y*,*z*_ are Pauli matrices, *m* is the effective mass term, and Δ ***k*** = ***k***−***k***_*K/K*′_ is the displacement of the wave vector ***k*** to the *K*/*K*′ valley in the momentum space. The broken parity-inversion symmetry of the unit cell introduces the effective mass term, and the mass term lifts the degenerated Dirac points and opens a band gap. As displayed in Figure 1(b), the first and second bands in red lines have a large gap when the length of the metallic pattern *p* = 6 mm, *q* = 4 mm. To obtain the synthetic gauge field, we construct an inhomogeneous SSP metasurface. We apply a linear gradient deformation in the *y* direction and translational invariance along the *x* direction, the length *p* of the *l*th row of the SSP structure is modulated linearly as

$$p_{l}=p_{1}+\left(p_{L}-p_{1}\right)\left(l-1\right)/\left(L-1\right)$$

where *p*_1_ and *p*_*L*_ are the length of the metallic pattern at the first and last layers of a gradient SSP structure. The *p*_1_ and *p*_*L*_ are fixed as 6 and 4 mm, respectively. The gradient $\sigma =\left(p_{L}-p_{1}\right)/\left(L-1\right)$ and the number of layers *L* are modulated. The length *q* has an inverted variation with *p*. The size of the first band gap ( $\Delta \omega =2\left|m\right|$ ) at *K*/*K*′ points has a linear relation with the coordinate *y*, as shown in Figure 1(c). Therefore, the effective mass term is linearly dependent to *y*, i.e. $m\left(y\right)=by$ . The insets illustrate the schematic of the unit cells in the top, middle and bottom layers of the SSP metasurface, and corresponding degenerated and opened band structures. The lower (upper) half part of the inhomogeneous SSP metasurface is defined as I (II), corresponding to *m* > 0 (*m* < 0). It indicates the vector potential *A* is introduced along the *z* direction with $A_{z}=m\left(y\right)$ , therefore the in-plane pseudo-magnetic field *B* represented by a yellow arrow in Figure 1(a) is constructed.

**Figure 2:**
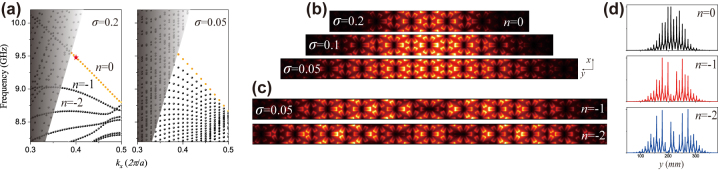
Chiral Landau level. (a) Quantized Landau level induced by the synthetic pseudo-magnetic field corresponding to the gradient *σ* = 0.2 (*L* = 11 layers) and 0.5 mm (*L* = 41 layers). (b) Electric field amplitude $\left|E_{z}\right|$ distribution of the chiral *n* = 0 Landau levels at *k* = 0.4(2*π*/*a*) for the different gradient *σ*. (c) $\left|E_{z}\right|$ distribution of *n* = −1 and −2 Landau levels at *k* = 0.4(2*π*/*a*) for the gradient *σ* = 0.05 mm. (d) Normalized $\left|E_{z}\right|$ as a function of *y* coordinate at *n* = 0, −1, and −2 Landau levels.

The artificial pseudo-magnetic field perpendicular to the *x*–*y* plane gives rise to discrete flat Landau levels. However, in our designed inhomogeneous SSP metasurface the in-plane pseudo-magnetic field results in the linear dispersion of the zeroth-order Landau level. The quantization of energy levels is expressed as [24], [34]:

$$\omega_{n}=\left\{\begin{array}{cc} \chi \mathrm{sign}\left(B_{x}\right)v_{D}k_{y},\; & n=0\\ \pm \sqrt{v_{D}^{2}k_{y}^{2}+2n\left|a\right|v_{D}},\; & n\neq 0 \end{array}\right.$$

where the *n* is the Landau-level index, the *χ* is the chirality corresponding to the *K* and *K*′ valleys, and *B* is the strength of the pseudo-magnetic field. The calculated chiral zeroth-order (*n* = 0) Landau level indicated by the yellow dots and discrete higher-order flat Landau levels are as displayed in Figure 1(d), when the gradient *σ* = 0.1 mm and the number of layers *L* = 21 along *y* direction. The chiral Landau level has linear dispersion and positive (negative) group velocity for *K* (*K*′) valley. The shaded regions represent line cones. The group velocity of the zeroth-order Landau level is independent of the strength of the pseudo-magnetic field related with the gradient *σ* directly. This linear dispersion has the slope *v*_*g*_ = ∂*ω*/∂*k* = −8.69 × 10^7^ m/s for *σ* = 0.1 mm which is same with those for *σ* = 0.2 (*L* = 11 layers) and 0.05 mm (*L* = 41 layers) as shown in Figure 2(a). The increasing gradient *σ* and the strength of the pseudo-magnetic field result in the increasing width of the adjacent Landau Levels. The chiral zeroth-order Landau level is bulk state and distributes in the large area in the middle of the inhomogeneous SSP metasurface, which is different from the topological edge state localized on the boundary of the quantum spin-Hall and valley-Hall systems. The eigenmode distribution of the chiral Landau level is controlled by the strength of the pseudo-magnetic field. As shown in Figure 2(b), the spreading area of the zeroth-order Landau level at the wave vector *k* = 0.4(2*π*/*a*) is decreasing corresponding to the gradient *σ* = 0.05, 0.1 and 0.2 mm, indicating that the stronger pseudo-magnetic field leads to the better confined electric field. Compared with the *n* = 0 Landau level concentrating on one region, the electric fields of *n* = −1 and −2 Landau levels distribute in two and three regions in the bulk respectively, as displayed in Figure 2(c) and (d). The positive high-order Landau levels exist within the light cone, and are not considered in the band dispersion (see the Supplementary Information).

**Figure 3:**
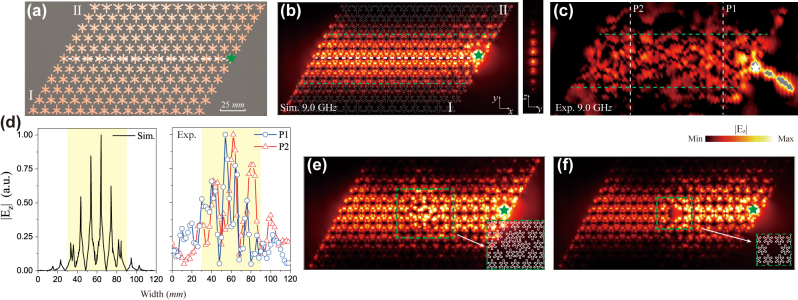
Experimental realization of a photonic Dirac waveguide. (a) Photograph of an inhomogeneous SSP metasurface sample with the gradient deformation of the metallic pattern. The area of the PCB sample is 252 × 118 mm^2^. The number of layers is *L* = 11 and the gradient *σ* = 0.2 mm. A white dashed line indicates the middle of the gradient SSP structure. (b) Left panel: simulated electric field of the chiral zeroth-order Landau level at frequency 9.0 GHz. Right panel: $\left|E_{z}\right|$ distribution in *y*–*z* plane. Two green lines highlight the region of the uniform distribution of the chiral Landau level. A green star marks the position of the excited source. (c) Experimental measurement of $\left|E_{z}\right|$ above the sample 1 mm. (d) Spatial amplitude of the electric field along the vertical dashed lines in (c). The simulated and experimental results demonstrate the chiral Landau level distributes in the large area about 30–90 mm. (e) and (f) Transport of the chiral Landau level at the position disorder and cavity defect, respectively.

The experiment is carried out to directly observe the chiral zeroth-order Landau level. The experimental sample of the SSP metasurface is implemented using a printed circuit board (PCB), in which the dielectric substrate is a high frequency dielectric material FR4 and the metallic pattern is made of thin copper layer, as shown in Figure 3(a). The area of the PCB is 252 × 118 mm^2^. The sample is placed on a piece of a thick foam board (*ɛ*_*r*_ ≈ 1), which ensures that the electromagnetic wave trapped on the surface of the SSP metasurface maintains symmetric along *z* direction. A long coaxial cable is attached on the sample and used as an excited source at the right terminal. A monopole antenna detects the electric field above the sample surface 1 mm. In order to effectively excite the electric field, we need to put the source at one arm of the snowflake pattern, which is consistent with the eigenmode distribution within one unit cell. The inhomogeneous SSP structure has 21 unit cells along *x* direction and 11 layers along *y* direction corresponding to the gradient *σ* = 0.2 mm, where the interface is defined as I–II type. The simulated and experimental electric fields of the chiral Landau level belong to *K*′ valley propagate towards the left as shown in Figure 3(b) and (c), which agree very well. The right propagation of the chiral Landau level can be excited when the source is placed at the leftmost end. In order to analyze the distribution character of chiral Landau level, the $\left|E_{z}\right|$ spatial amplitudes marked by two vertical dashed lines in Figure 3(c) are plotted as a function of *y* coordinate in Figure 3(d). It can be observed that the chiral Landau level is not confined on the interface and uniformly distributes in the large area. The chiral Landau level remains robust when the small disorder is introduced to the position of metallic patterns in the middle of the SSP structure as shown in Figure 3(e). However, the large cavity defect has non-negligible impact on the transport of the chiral Landau level, displayed in Figure 3(f).

**Figure 4:**
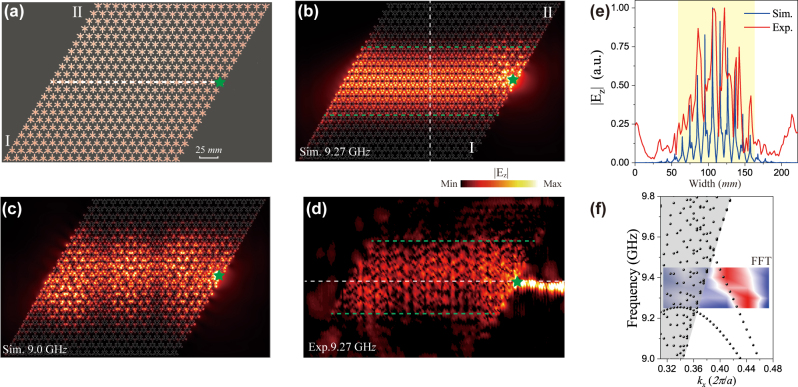
Straight photonic Dirac waveguide. (a) Photograph of the photonic straight Dirac waveguide composed of the inhomogeneous SSP metasurface sample with the gradient *σ* = 0.1 and the number of layers *L* = 21. The area of the rectangular sample is 372 × 222 mm^2^. (b) Simulated electric field of the chiral zeroth-order Landau level at frequency 9.27 GHz. (c) Simulated $\left|E_{z}\right|$ distribution of *n* = 0 and −1 Landau levels excited at frequency 9.0 GHz simultaneously. (d) Experimental measurement of $\left|E_{z}\right|$ at frequency 9.27 GHz in the Dirac waveguide. (e) Simulated and experimental result of $\left|E_{z}\right|$ along the vertical dashed line in (b). (f) Measured the band dispersion of the chiral Landau level by applying the discrete Fourier transform.

The photonic straight Dirac waveguide is demonstrated in our proposed inhomogeneous SSP metasurface, which possesses controllable artificial pseudo-magnetic field. Figure 4(a) shows the experimental sample with the gradient *σ* = 0.1 mm and *L* = 21 layers, which has the larger area of the electric field distribution compared with the structure with *σ* = 0.2 mm. The $\left|E_{z}\right|$ approximately distributes at 60–160 mm in the simulation and experiment as displayed in Figure 4(b) and (d), and highlighted in yellow in Figure 4(e), It obviously reveals that the Dirac waveguide has high capacity of the energy transport based on the chiral Landau level. The transport region is manipulated by the strength of the pseudo-magnetic field. At the lower frequency 9.0 GHz, the *n* = 0 and *n* = −1 Landau levels are simultaneously excited, and the electric field has non-uniform distribution, as shown in Figure 4(c). By applying the discrete Fourier transformation along the white dashed line in Figure 4(d), we obtain the experimental dispersion relation of the chiral Landau level, which has a good agreement with the calculated band dispersion with the negative group velocity in Figure 1(d). When the excited antenna is put at the left (right) terminal, the Dirac waveguide at the *K* (*K*′) valley is well excited.

**Figure 5:**
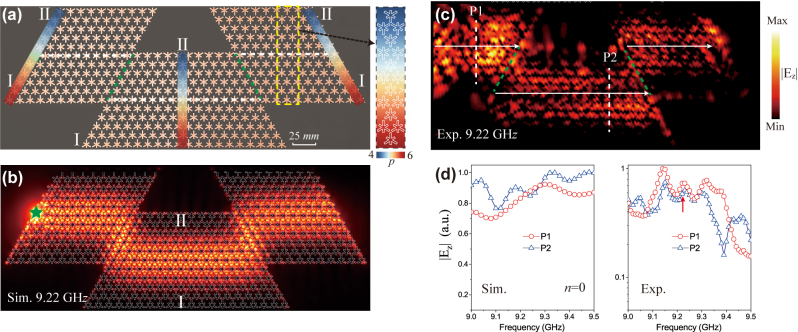
Bending photonic Dirac waveguide. (a) Photograph of a Ω-shaped Dirac waveguide with three inhomogeneous SSP structures at the gradient *σ* = 0.2 mm and *L* = 11 layers. Right panel: the schematic of the gradient. The whole area of the structure is 432 × 170 mm^2^. (b) and (c) Simulated and experimentally measured transport of the guided mode in the Dirac waveguide at frequency 9.22 GHz. (d) Simulated and measured $\left|E_{z}\right|$ integration at P1 and P2 ports corresponding to the chiral Landau level at 9.0–9.5 GHz.

We further design a Dirac waveguide with Ω-shape. The experimental configuration is shown in Figure 5(a), which consists of three inhomogeneous SSP structures at the gradient *σ* = 0.2 mm. The Dirac waveguide has 60° and 120° bends at two corners, and three regions all have the interface with I-II type. The top layer of the middle region has ten unit cells to avoid the coupling of bulk states between the left and right regions. The simulated and experimental $\left|E_{z}\right|$ distributions are shown in Figure 5(b) and (c), in which the white arrows denote the transport of the chiral Landau level in the Dirac waveguide. The guided mode at the K valley launched from the left terminal propagates towards the right, and couples and excites the bulk state in the middle and right SSP structures. There are no observable scattering losses at two bends. We experimentally measure the integration of the electric field amplitude at the input (P1) and output (P2) ports indicated by two dashed lines in Figure 5(c). The integration of $\left|E_{z}\right|$ at a specialized frequency range is shown in Figure 5(d), corresponding to the *n* = 0 Landau level, which is based on the calculated band dispersion and simulated transmission spectrum.

**Figure 6:**
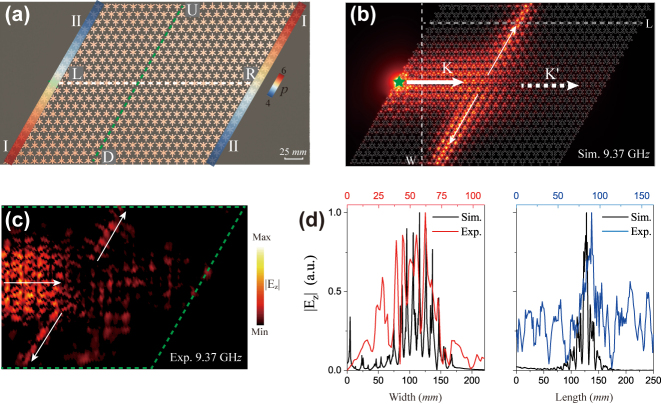
Beam splitter composed of photonic Dirac waveguides. (a) Experimental sample of the designed beam splitter by using the Dirac waveguide and the topological edge state. The device has two inverted gradient SSP structures with *σ* = 0.1 mm and layers *L* = 21. The area of the sample is 420 × 222 mm^2^. (b) and (c) Simulated and measured transport of the beam splitting at 9.37 GHz. White arrows indicate the propagating direction of the bulk and edge states. (d) $\left|E_{z}\right|$ along the horizontal and vertical directions indicated by dashed lines in (b), corresponding to the Dirac waveguide and the topological edge state respectively.

The beam splitter is designed to fabricate a photonic device consisting of the Dirac bulk state and edge state waveguides. The rhombus-shaped experimental sample is shown in Figure 6(a). The device is composed of two inverted gradient SSP structures with *σ* = 0.1 mm and layer *L* = 21, in which four channels intersect obliquely and separate the structure into four domains. The left (L) and right (R) channels are I–II and II–I types respectively, supporting the bulk state of the chiral zeroth-order Landau level, while the upper (U) and downward (D) channels transport edge state confined on the interfaces. As displayed in Figure 6(b) and (c), the excited guided mode at the *K* valley travels from the left channel to the upper and downward channels, but is nearly suppressed on the right channel. The transmission partition of the edge state is dependent on the geometry at the topological channel intersection, which has been achieved in the topological valley-Hall system by manipulating valley pseudospins [45], [46]. The transmitted energy proportion of upper and downward channels is unequal in a topological valley system. Owing to the large-area transport of the zeroth-order Landau level with high-capacity energy, the transmission proportion of two edge state channels is equal and approximately 0.5. The partition phenomenon can be used to design a beam splitter in the application to manipulate the electromagnetic wave. The measured $\left|E_{z}\right|$ distributions along the horizontal and vertical directions as displayed in Figure 6(d) demonstrate the Dirac bulk state with large area and the confined edge state in the transport channels, which also agree well with simulated results.

## 3 Conclusions

In this work, we experimentally realize an inhomogeneous SSP metasurface with an artificial in-plane pseudo-magnetic field. The spatially variable mass term gives rise to the pseudo-magnetic field, and is manipulated by the linear variation of the bandgap width of the Dirac cones. The chiral zeroth-order Landau level induced by the strong pseudo-magnetic field is one-way propagative bulk state, and can be utilized to realize the photonic straight Dirac waveguide with the large-area distribution of the bulk mode. The electromagnetic mode in the Dirac waveguide is dependent with the gradient deformation of SSP metasurface i.e. the strength of the pseudo-magnetic field, and support high-capacity energy transport. The Ω-shaped Dirac waveguide is also obtained in the experiment, and the zeroth and high order Landau levels are measured. We combine the transport of the bulk mode in a Dirac waveguide and the topological edge state in a valley photonic structure, and thus experimentally obtain a beam splitter with equal energy partition in two output ports. Our proposed SSP metasurface provides a route for generating an artificial in-plane magnetic field, and an ideal platform for manipulating surface wave to design more photonic devices in future applications.

## Supplementary Material

Supplementary Material Details
