# Supplementary material for: Photonic Dirac waveguide in inhomogeneous spoof surface plasmonic metasurfaces
Source: Nanophotonics. 2024 Jul 11;13(20):3847–54. doi: 10.1515/nanoph-2024-0200 (PMC11465999; doi:10.1515/nanoph-2024-0200)
Supplement: Supplementary file 1 — Supplementary Material Details [file j_nanoph-2024-0200_suppl_001.docx]

**Supplementary Information for****Photonic Dirac waveguide in i****nhomogeneous spoof surface plasmonic metasurfaces**

Yuting Yang^1,2 *^, Juyi Zhang^1^, Bin Yang^1^, Shiyu Liu^1^, Wenjie Zhang^1^, Xiaopeng Shen^1^, Liwei Shi^1^ and Zhi Hong Hang ^3^^,4^

*^1^School of Materials and Physics, China University of Mining and Technology, Xuzhou 221116, China*

*^2^State Key Laboratory of Millimeter Waves, Southeast University, Nanjing 210096, China*

*^3^School of Physical Science and Technology & Collaborative Innovation Center of Suzhou Nano Science and Technology, Soochow University, Suzhou 215006, China*

*^4^Institute for Advanced Study, Soochow University, Suzhou 215006, China*

* *Corresponding author: yangyt@cumt.edu.cn*

The designed inhomogeneous spoof surface plasmonic (SSP) metasurfaces in our work have *n*=0 chiral Landau level and other higher-order Landau levels. The band diagram in Figs. 1(d) and 2(a) only displays the negative Landau levels. The positive high-order Landau levels exist within the light cone, and are not considered in the band dispersion. The Landau levels are in narrow range of the wave vectors, as shown in Fig. S2(a). To well illustrate this issue, we study the photonic Dirac waveguide in a two-dimensional valley photonic crystal. As shown in Fig. S1, the reduced symmetry of the photonic crystal by rotating a triangular pillar leads to the broken Dirac cones at K and K’ valleys [1,2]. The proposed gradient photonic crystal also possesses chiral Landau levels. The red dashed line in Fig. S1(c) indicates the location of the light line. Compared with the band dispersion of the two-dimensional photonic crystal, the SSP metasurface can not clearly display positive Landau levels. The positive higher-order Landau level can be found in the bulk band. As shown in Fig. S2(b) and S2(c), the *n*=1 and *n*=2 Landau levels appear at 10.68 and 10.78 GHz at the wave vector *k*=0.4 (2*π*/*a*).


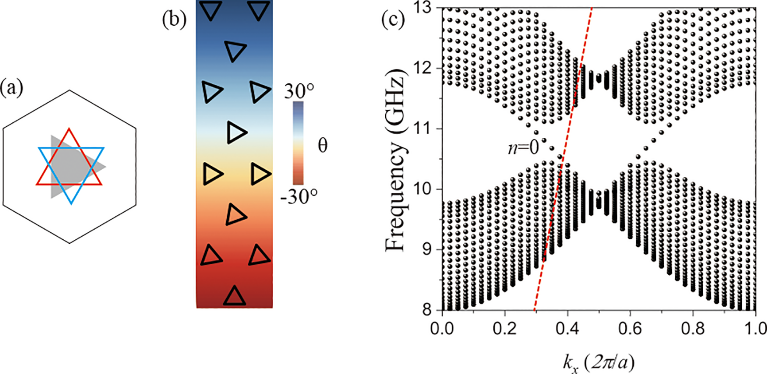


**Figure S1:** (a) Schematic of the unit cell of the two-dimensional photonic crystal. The lattice constant is *a* =22 *mm*, and the length of the triangular metallic pillar embedded in the air background is L=9.5 *mm*. (b) Schematic of the designed gradient photonic crystal. (c) Band dispersion of the chiral Landau level in the photonic crystal.


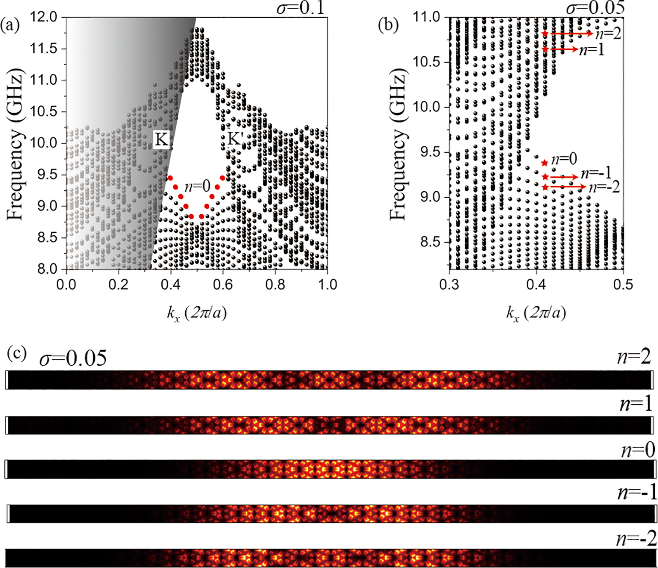


**Figure S2:** (a) and (b) Quantized Landau level induced by the synthetic pseudo-magnetic field corresponding to the gradient *σ*=0.1 (L=21 layers) and 0.5 *mm* (L=41 layers) in inhomogeneous SSP metasurfaces. (c) Eigenmode distribution of electric fields for *n*=-2, -1, 0, 1 and 2 Landau level corresponding to 9.15, 9.28 9.45, 10.68 and 10.78 GHz, respectively.

**References**

[1] Lu, J., Qiu, C., Ke, M. & Liu, Z. Valley Vortex States in Sonic Crystals. Phys Rev Lett 116, 093901 (2016).

[2] Ye, L., Yang, Y., Hang, Z. H., Qiu, C. & Liu, Z. Observation of valley-selective microwave transport in photonic crystals. Appl. Phys. Lett. 111, 251107 (2017).
